# Management of Immunity Alteration-Induced Chronic Pain During the Coronavirus Disease-2019 (COVID-19) Pandemic

**DOI:** 10.3389/fmicb.2020.572318

**Published:** 2020-09-24

**Authors:** Wuping Sun, Hong Gao, Yuhui Luo, Hushan Zheng, Xiang Liao, Donglin Xiong, Lizu Xiao

**Affiliations:** ^1^Department of Pain Medicine and Shenzhen Municipal Key Laboratory for Pain Medicine, Shenzhen Nanshan People's Hospital and the 6th Affiliated Hospital of Shenzhen University Health Science Center, Shenzhen, China; ^2^National Key Clinical Pain Medicine of China, Shenzhen, China

**Keywords:** COVID-19, chronic pain, neuropathic pain, SARS-CoV-2, pain management, coronavirus

## SARS-CoV-2 & COVID-19

Coronavirus disease-2019 (COVID-19), caused by severe acute respiratory syndrome coronavirus 2 (SARS-CoV-2), which was identified at the end of 2019, has become a widespread, global public health crisis, raising many concerns (Khan et al., 2020). According to the newest data from the World Health Organization (WHO), global SARS-CoV-2 infection cases have reached 18.6 million, with the reported deaths of more than 700,000 individuals (WHO, 2020).

Coronaviruses are enveloped, positive-stranded RNA viruses, with an average diameter of 100 nm. The genome length of most coronaviruses ranges from 26 to 32 kb. Coronaviruses can infect various vertebrate species, including bats, dogs, and humans (Fan et al., 2019; Sit et al., 2020). Since the start of the Twenty one century, humans have encountered coronavirus outbreaks three times: severe acute respiratory syndrome-associated coronavirus (SARS-CoV-1), in 2003 (de Wit et al., 2016), Middle East respiratory syndrome-related coronavirus (MERS-CoV), in 2008 (Hemida et al., 2020), and SARS-CoV-2, in 2019 (Wu F. et al., 2020). Genetic comparisons have indicated 79.5% similarity between SARS-CoV-2 and SARS-CoV, and the similarity is up to 96% between SARS-CoV-2 and a coronavirus strain isolated from bats (Zhou et al., 2020).

According to the structural and genome-wide association studies, SARS-CoV-2 can more easily infect and replicate in host cells than other coronaviruses (Gussow et al., 2020; Yan et al., 2020). SARS-CoV-2 infections are responsible for the ongoing, global COVID-19 pandemic, which has a fatality rate between 2 and 4% (Weiss and Murdoch, 2020). SARS-CoV-2 infections result in major impacts on the human respiratory system (Rothan and Byrareddy, 2020), with fever, mild cough, pneumonia, and dyspnoea (Singhal, 2020). COVID-19 patients also experience various neurological symptoms, such as headache, epilepsy, disturbed consciousness (Mao et al., 2020; Wu Y. et al., 2020), smell, vision and taste loss, neuralgia (Jin et al., 2020; Mao et al., 2020), and acute neurological disorders, such as stroke and seizure (Jin et al., 2020) in COVID-19 patients. It has been reported that SARS-CoV-2 infection affected the central and peripheral nervous system manifesting as dysosmia, visual disturbances, and neuralgia after (Mao et al., 2020). Abdelnour et al. has reported a case of SARS-CoV-2 infection manifesting as peripheral neuropathy (Abdelnour et al., 2020). This evidence demonstrates that SARS-CoV-2 can cause serious malfunction and damage to both the central and peripheral nervous systems.

## SARS-CoV-2 Infections in Individuals With Altered Immunity

It has been reported that SARS-CoV-2 infections in elderly and weak individuals cause more severe syndromes, such as acute respiratory distress syndrome (ARDS) and acute lung injury (ALI), which are associated with lung malfunction and death (Matuschak and Lechner, 2010). Patients with chronic obstructive pulmonary disease (COPD) and smoking history have also been reported to experience worse progression and outcomes when diagnosed with COVID-19 (Zhao et al., 2020). COVID-19 in cancer patients has a 36% lethality, and worse prognosis has been observed among older individuals (Stroppa et al., 2020).

Human immunodeficiency virus (HIV)-positive patients may be more vulnerable to COVID-19 due to their immune-compromised status. A recent clinical observation reported that COVID-19 results in a death rate of approximately 9% among patients living with HIV (Harter et al., 2020). Besides, some patients treated with opioids could be more susceptible to SARS-CoV-2 infections because treatments with morphine and fentanyl have been reported to be the most immunosuppressive (Mellon and Bayer, 1998; Shavit et al., 2004). Opioids act on the hypothalamic-pituitary-adrenal (HPA) axis and the autonomic nervous system, further suppressing the immune system (Mellon and Bayer, 1998; Shavit et al., 2004; Plein and Rittner, 2018). These studies suggest that hypo-immune individuals might have higher risks and worse outcomes associated with COVID-19.

On the other hand, the possibility of SARS-CoV-2 infection in hyper-immune individuals is also worldwide discussed because of their immunological background and therapies. It has been reported that hyper-immunity individuals have received treatment with immunosuppressive or modulatory agents; these approaches may increase the possibility of SARS-CoV-2 infection (Cai et al., 2020). Therefore, individuals with altered immunity (hypo-immune & hyper-immune) may require additional attention to prevent the infection of SARS-CoV-2 during the COVID-19 outbreak.

## SARS-CoV-2 Infection-Induced Immune Alteration

It is known that the induction of a cytokine storm is the basic cause of pathogenic inflammation in COVID-19 (Jose and Manuel, 2020; Mehta et al., 2020). Cytokine storm is an acute hyperinflammatory response responsible for critical illness in many conditions, including viral infections, cancer, sepsis, and multi-organ failure (Bhaskar et al., 2020). The elevation of cytokines in the blood is crucial to induce cytokine storm and immunosuppression in the transition of severity in COVID-19 patients (Bhaskar et al., 2020). Laboratory results have shown that dysregulation in the immune system has occurred in COVID-19 patients. SARS-CoV-2 infection increases the plasmatic secretion of interleukin 1β, interferon-γ, interferon-γ-induced proteins, monocyte chemoattractant protein-1, IL-4, and IL-10 (Vinciguerra et al., 2020). Moreover, it has been reported that SARS-CoV-2 infection induces the up-regulation of a series of interferon-stimulated genes, indicative of immune and interferon responses to the virus (Blanco-Melo et al., 2020). These results demonstrated that SARS-CoV-2 infection-induced immune alteration in COVID-19 patients.

## Immunity Alteration-Induced Chronic Pain

The SARS-CoV-2 infection causes systemic inflammation and dysregulation of immunity, resulting in various delayed neurological complications. Both central and peripheral nervous systems have been reported to be involved in the immune-mediated manifestations in COVID-19 patients (Abdelnour et al., 2020; Mao et al., 2020). It is known that the virus could enter the brain carried by infected immune cells (Bergmann et al., 2006). Several regions in the brain, including vasculature, meninges, and choroid plexus, could be the entry sites for virus-infected immune cells (Engelhardt et al., 2017). Besides, SARS-CoV-2-induced cytokines, IL-6, IL-1β, TNF, and IL-17 could facilitate the entry of the virus into the brain through disrupting the blood-brain barriers (BBB) (Erickson and Banks, 2018). On the other hand, the hypothalamus in the brain also contributes to the dysregulation of immunity. IL-6, IL-1β, and TNF-α have been reported to be robust activators of the HPA axis (Dantzer, 2018). HPA axis plays a central role in regulating systemic immunity and is majorly activated by BBB dysfunction and neurovascular inflammation (Dantzer, 2018). These studies suggested that SARS-CoV-2 infection-induced immune alteration could further result in the concurrence of chronic pain since it affects the nervous system.

Chronic pain is a complex and distressing problem, which significantly impacts the life quality of each individual. Chronic pain is not merely an accompanying symptom and can be associated with various underlying causes, including immunity alterations, and viral infections. Patients with chronic pain must be given effective and continuous treatments to manage their long-term pain at an acceptable level. To date, the prevalence of COVID-19 appears to continue increasing exponentially worldwide. Constant exposure risk to COVID-19 is currently expected to become the new normal. In this situation, the management of immunity alteration-induced chronic pain may require additional attention. Here we summarize several types of chronic pain, which are closely related to the alteration of immunity in individuals, and give some recommendations from a clinical view.

## Chronic Pain in Hypo-Immunity Individuals

### Varicella-Zoster Virus Infection-Induced Neuropathic Pain

HZ is an acute, cutaneous viral infection caused by the reactivation of the VZV (Saguil et al., 2017). The incidence of HZ has been estimated to be 7.7% in China, with a lifetime risk of 30% in each individual (Yang et al., 2019). The risk factors associated with the reactivation of VZV have not yet been clarified; however, malignancies, immune deficiencies, solid organ, and bone marrow transplantations, autoimmune diseases, psychological conditions, emotional stress, and immunosuppressive therapies have been identified as possible major risk factors (Wei et al., 2019). In hypo-immune conditions, especially those associated with decreased cell-mediated immune status, the risk of VZV reactivation is 100-fold higher than in healthy subjects (Singh et al., 2020). It has been reported that SARS-CoV-2 infected individuals have also developed severe acute herpetic neuralgia despite the early initiation of antiviral therapy (Saati et al., 2020; Shors, 2020). Herpes zoster (HZ) might be an indicator of latent SARS-CoV-2 infection because the clinical presentation of HZ even in patients having mild or no upper respiratory symptoms should be considered as an alarming sign for SARS-CoV-2 infection (Elsaie et al., 2020), which highlights the possibility of COVID-19-induced HZ.

Postherpetic neuralgia (PHN) is the most common complication in approximately one-fifth of HZ patients, especially among elderly individuals (Gershon et al., 2010). PHN is defined as skin-distributed pain that persists for at least 3 months after acute HZ (Salvetti et al., 2019). The treatment for PHN focuses on symptom control, including the use of topical lidocaine or capsaicin and oral gabapentin, pregabalin, or tricyclic antidepressants (Hempstead et al., 2020; Kopel and Brower, 2020), as well as the improvement of immunity and antiviral treatments (Huning et al., 2019; Hunter et al., 2020). The analgesic effects of traditional treatments have not been reported to have good efficacy among PHN patients with older age, serious skin lesions, or long disease courses. Neuromodulation, such as spinal cord stimulation, may represent a new approach for pain relief among these patients (Huang et al., 2020).

### HIV Infection-Induced Neuropathic Pain

Acquired immune deficiency syndrome (AIDS) is associated with various infection symptoms, and peripheral neuropathic pain is the most common and severe neurological manifestation that has been reported in HIV-positive, immunocompromised individuals (Amaniti et al., 2019). Statistical analysis has revealed that up to one-third of HIV-infected individuals suffer from neuropathic pain, which presents as distal, symmetrical, axonal, and peripheral sensory neuropathic pain, accompanied by a burning sensation and paraesthesia, which primarily affects the legs and hands (Gabbai et al., 2013). The possible pathogenesis of HIV infection-induced neuropathic pain includes tumor necrosis factor-α (TNF-α) (Zheng et al., 2011), CCAAT/Enhancer Binding Protein β (CEBPβ) phosphorylation (Yi et al., 2018), and mitochondrial oxidative stress (Kanda et al., 2016). The current clinical treatment for HIV-induced neuropathic pain includes nonopioid pain relievers, opioid analgesics, adjuvant medications, and psychosocial therapies (Krashin et al., 2012).

## Chronic Pain in Hyper-Immunity Individuals

### Rheumatoid Arthritis

Reactive arthritis (RA), an autoimmune disease, is the most common chronic inflammatory disease. RA is characterized by the progressive, symmetric inflammation of affected joints and tendon (tenosynovitis), resulting in both cartilage destruction and bone erosion (Lin et al., 2020). The clinical manifestations of RA vary greatly among individuals and can become more severe without medical intervention. The prevalence of RA has been reported to range from 4 to 13 per 1,000 individuals, and the risk factors include age, gender, genetics, smoking, obesity, exposure to ultraviolet (UV) light, drugs, changes in the microbiome of the gut, mouth, and lungs, periodontal disease (periodontitis), and infections (Deane et al., 2017). Besides, SARS-CoV-2 infection also causes RA (Ono et al., 2020). Joint inflammation in RA is mediated by T-cells, B-cells, macrophages, fibroblasts, and inflammatory cytokines (Lin et al., 2020). Currently available therapeutic drugs include the administration of non-steroidal anti-inflammatory drugs (NSAIDs), immunosuppressive glucocorticoids (Hajialilo et al., 2016), and disease-modifying anti-rheumatic drugs (DMARDs) (Fries, 2000).

### Ankylosing Spondylitis

Ankylosing spondylitis (AS) is both an autoimmune rheumatological arthritis and a chronic inflammatory disease. AS is reported to be highly correlated with the presence of human leukocyte antigen (HLA)-B27 (Hill et al., 1976). Moreover, genome-wide association studies (GWASs) have identified numerous single-nucleotide polymorphisms (SNPs) related to AS susceptibilities, such as those in *IL-23R, IL-17A, RUNX3*, and *BCL11B* (Liu et al., 2020). AS is caused by chronic inflammatory disorders, manifested as structural damage to the spinal and sacroiliac joints, which subsequently develops into ankylosis, due to new osteogenesis (Sieper and Poddubnyy, 2017). The clinical symptoms of AS include chronic back pain, morning stiffness, fatigue, and the loss of spinal mobility (Sieper and Poddubnyy, 2017). The clinical treatment of AS typically involves suppressing immunity and anti-inflammatory medications (Yang et al., 2018). NSAIDs are the first-line drugs used clinically and are considered the most effective therapeutic approach, according to current management recommendations (Ward et al., 2019).

### COVID-19-Induced Psychological Stress Associated With Chronic Pain Management

Chronic pain involves complex brain circuits, which include sensory, emotional, cognitive, and interoceptive processing (Simons et al., 2014). An association exists between psychosocial factors and the severity of chronic pain. Psychosocial factors have been reported to affect the development of chronic pain, as well as pain treatment outcomes. The severity of chronic pain can be evaluated by pain-related distress. COVID-19 has caused an international public health emergency and poses a tremendous challenge to psychological resilience. These adverse psychological impacts and psychiatric symptoms include depression, anxiety, panic, somatic symptoms, self-blame, guilt, post-traumatic stress disorder (PTSD), delirium, psychosis, and even suicide (Steenblock et al., 2020). A recent survey has revealed that 12.5, 37.8, and 36.4% of participants reported sleep difficulties, paranoia regarding the acquisition of COVID-19 infection, and distress related to social media. A perceived mental healthcare need was reported for more than 80% of participants (Roy et al., 2020). These phycological challenges could affect the management of chronic pain. Abnormal psychological status results in more severe chronic pain and increased difficulty experiencing clinical relief.

Moreover, the reasons for adverse psychological outcomes among patients ranged from inadequate access to personal protective equipment, shocking news media, feeling not supported, helpless and hopeless, experiencing insomnia, undergoing complicated medical procedures and environments, and the relatively high infection rate among medical staff and patients (Spoorthy et al., 2020). Therefore, phycological counseling became critical for the management of chronic pain during the COVID-19 outbreak. Cognitive-behavioral therapy is recommended as a psychological intervention for patients with chronic pain. Strengthening the patient's confidence could be necessary to relieve psychological stress in chronic pain patients during the COVID-19 outbreak.

### COVID-19 Situational Responses for Chronic Pain Management

1. Postpone immunosuppressive therapy for patients with immunity alteration-induced chronic pain.

The postponement or stoppage of immunosuppressive therapy, such as immunosuppressive glucocorticoids, should be considered unless the pain condition does not allow these options. This suggestion is as same as the international psoriasis council (IPC) statement on the Coronavirus (COVID-19) Outbreak. Patients using steroids, azathioprine, methotrexate, and cyclosporine should pay additional attention to the proper use of personal protective equipment.

2. Psychological counseling for patients with chronic pain

Psychological evaluations are necessary for patients with chronic pain, especially during the COVID-19 outbreak. Cognitive-behavioral therapy is recommended as a psychological intervention for patients with chronic pain. Strengthening the patient's confidence is recommended to relieve psychological stress in chronic pain patients during the COVID-19 outbreak.

3. Remote pain management, telemedicine, and medical quarantine for patients with chronic pain

Blood tests and instrumental examination interventions, such as functional MRI (fMRI), electroencephalogram (EEG), nerve block, and spinal cord stimulation (SCS), should be avoided unless critically necessary during the COVID-19 outbreak. Patients should medically quarantine themselves, and avoid contact with other individuals, except in cases of emergency. Clinical investigations and medical interventions that require repeated access to hospitals or medical staff may increase the susceptibility to SARS-CoV-2 infection. Outpatient visits and patient treatments should be performed with care to avoid potential infections within the hospital setting. If possible, patients' pain conditions should be monitored remotely or through telemedicine, to minimize the risks of SARS-CoV-2 infection. Therefore, clinical investigations and medical interventions should be suspended or postponed.

4. Monitoring body temperature, wearing masks, and avoid contact with outside individuals for inpatients should be given during the COVID-19 outbreak

For inpatients, a body temperature monitor strategy could avoid the internal infection of SARS-CoV-2 in the hospital. Wearing masks is the basic prerequisite for inpatients. A medical staff has the right to reject patients who do not wear a mask and ask inpatients to follow the local instructions and guidelines to avoid contact with outside individuals during the COVID-19 outbreak.

5. Special care for inpatient should be given to prevent the infection of SARS-CoV-2

Patients in an emergency or eager for treatment in the hospital have to receive SARS-CoV-2 nuclear acid assay and CT image check before entering into the hospital. For those suspected COVID-19 patients, if medical treatment is critically necessary, medical staff has to make adequate preparation to protect themselves and patients. Besides, disposable materials, including masks and gloves, should not be used repeatedly, and particular attention should be paid to cleaning and sterilization. All procedures must follow local instructions and guidelines.

## Concluding Remarks

The pandemic of COVID-19 has made great challenges to the social, medical system, particularly in light of the redistribution of medical staff, beds, equipment, and resources against SARS-CoV-2 infection. Chronic pain is suffering, significantly impacts the quality of life. Chronic pain patients have received limited treatment and discounted services during the COVID-19 outbreak due to limit the spread of SARS-CoV-2 infection. Chronic pain patients may also have increased infection risks to SARS-CoV-2 due to complicated reasons. Therefore, professional management of immune modulation-induced chronic pain during the COVID-19 outbreak is critical. In this opinion, we provide several recommendations, which represent the best available strategy and expert opinion at present, to aid the healthcare of those with chronic pain. However, our recommendations may need an update to adapting the local instructions and guidelines.

## Author Contributions

WS, HG, YL, HZ, XL, DX, and LX were responsible for the concept and design of the study. WS, DX, and LX performed writing of the manuscript. WS obtained funding. All authors contributed to the article and approved the submitted version.

## Conflict of Interest

The authors declare that the research was conducted in the absence of any commercial or financial relationships that could be construed as a potential conflict of interest.
